# Dilead(II) oxyhydroxide bromide

**DOI:** 10.1107/S241431462600790X

**Published:** 2026-08-07

**Authors:** Hibiki Kunisawa, Jun-ichi Yamaura, Toshihiro Nomura

**Affiliations:** a Department of Physics, Shizuoka University, Shizuoka 422-8529, Japan; b Institute for Solid State Physics, The University of Tokyo, Kashiwa, Chiba, 277-8581, Japan; Vienna University of Technology, Austria

**Keywords:** crystal structure, Pb(II), oxyhydroxide, bromide, square anti­prismatic coordination

## Abstract

The crystal structure of the title compound, Pb_2_O(OH)Br, has been determined by single-crystal X-ray diffraction. The compound crystallizes in the tetra­gonal space group *P*4_2_/*mcm*. The structure consists of [PbO_4_Br_4_] square anti­prisms, which form square layers in the *ab* plane and are stacked alternately along the *c-*axis direction. The hydroxide site is assigned on the basis of bond-valence-sum calculations.

## Abstract 2

The crystal structure of Pb_2_O(OH)Br has tetra­gonal symmetry and differs from those of the Cl (ortho­rhom­bic) and I (monoclinic) analogues.

## Graphical abstract

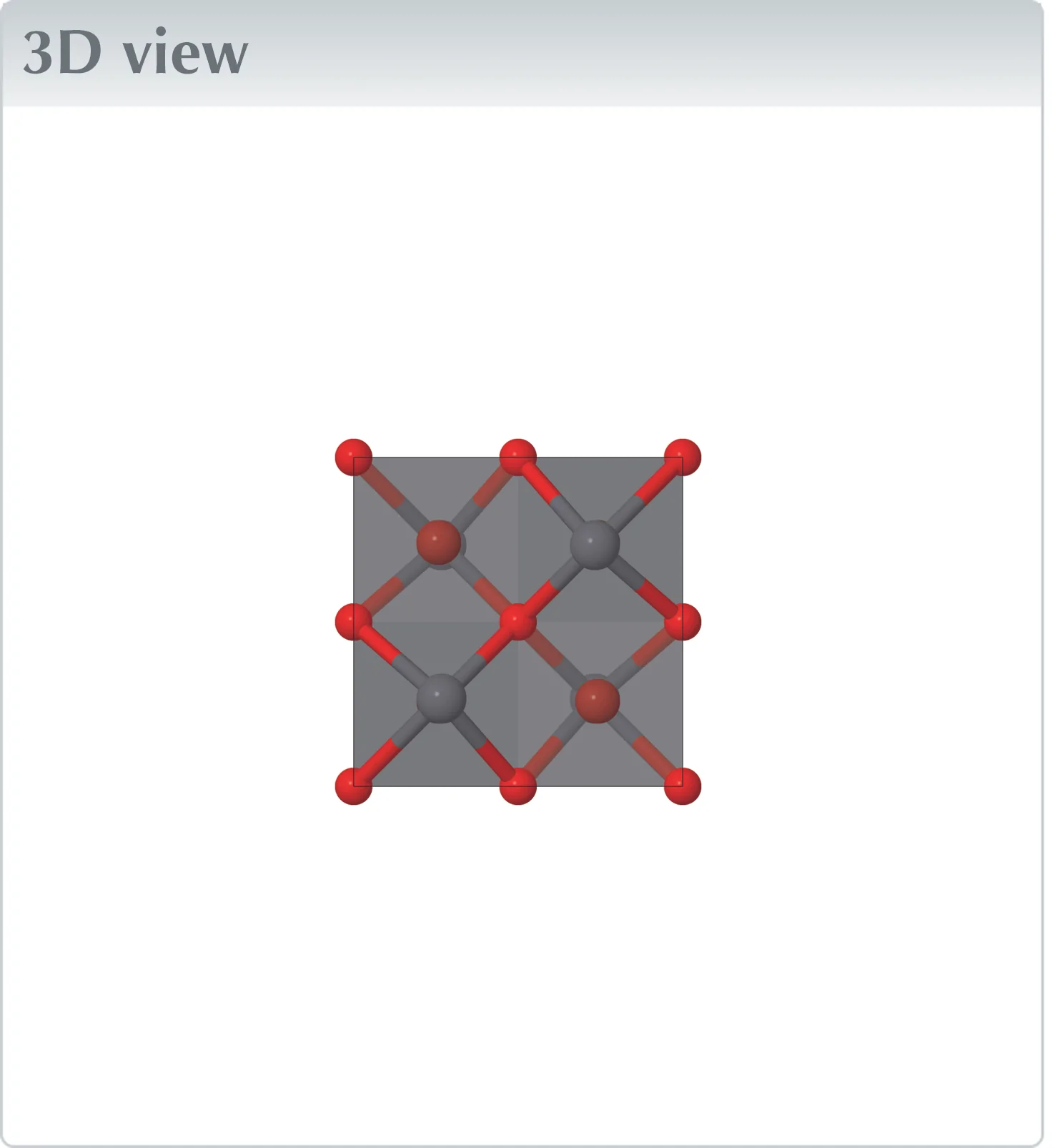

## Structure description

Lead oxyhalides comprise a diverse family of compounds with a variety of crystal structures. Among them, Pb_2_O(OH)Cl (Turner *et al.*, 2015 ▸), Pb_2_O(OH)I (Siidra *et al.*, 2013 ▸), and Pb_3_O_2_(OH)Br (Krivovichev & Burns, 2001 ▸) are oxide–hydroxide halides related to the title compound. In this work, we report the crystal structure of Pb_2_O(OH)Br determined by single-crystal X-ray diffraction.

**Figure 1:**
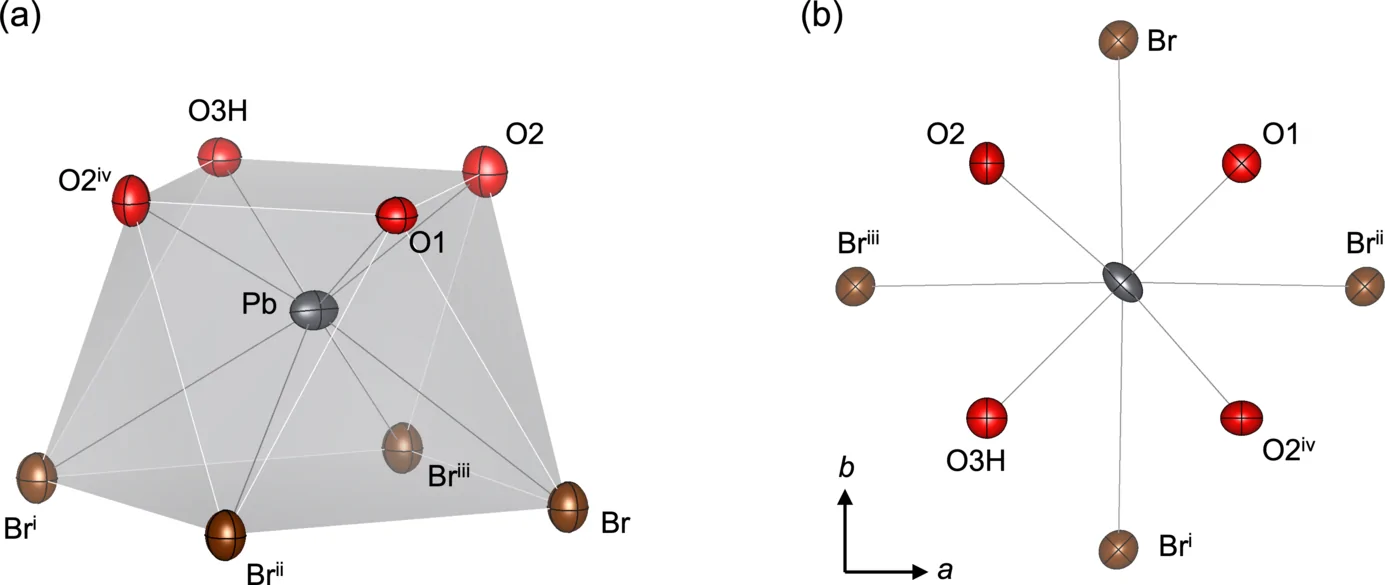
(*a*) Coordination environment of the Pb atom. Displacement ellipsoids are drawn at the 50% probability level; (*b*) the [PbBr_4_O_4_] coordination polyhedron viewed along the *c* axis. [Symmetry codes: (i) *x*, *y* − 1, *z*; (ii) −*x* + 1, −*y* + 1, −*z*; (iii) −*x*, −*y* + 1, −*z*; (iv) *y*, −*x*, −*z* + 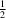.]

**Figure 2:**
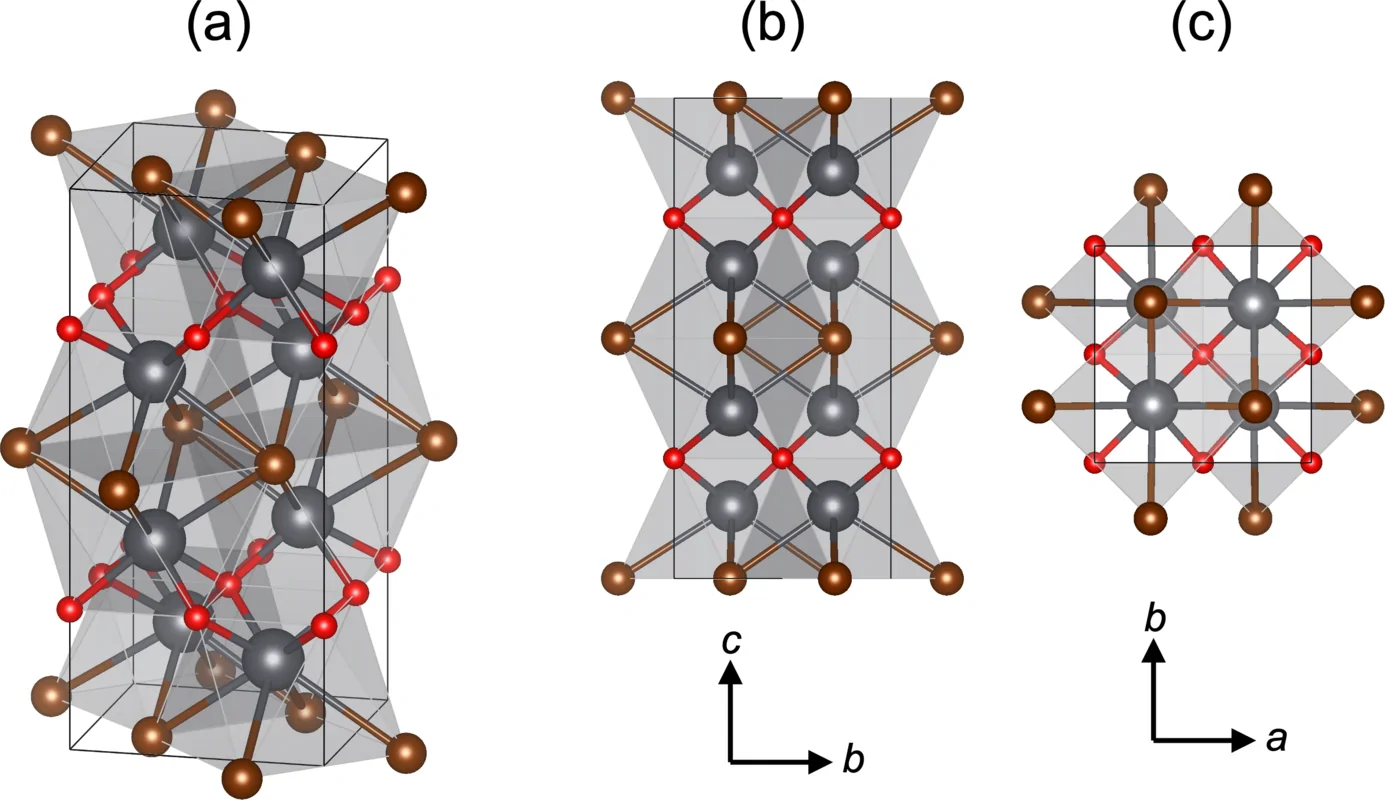
The crystal structure of Pb_2_O(OH)Br with polyhedral representation. (*a*) Perspective view, projections along (*b*) the *a* and (*c*) the *c* axes.

**Figure 3:**
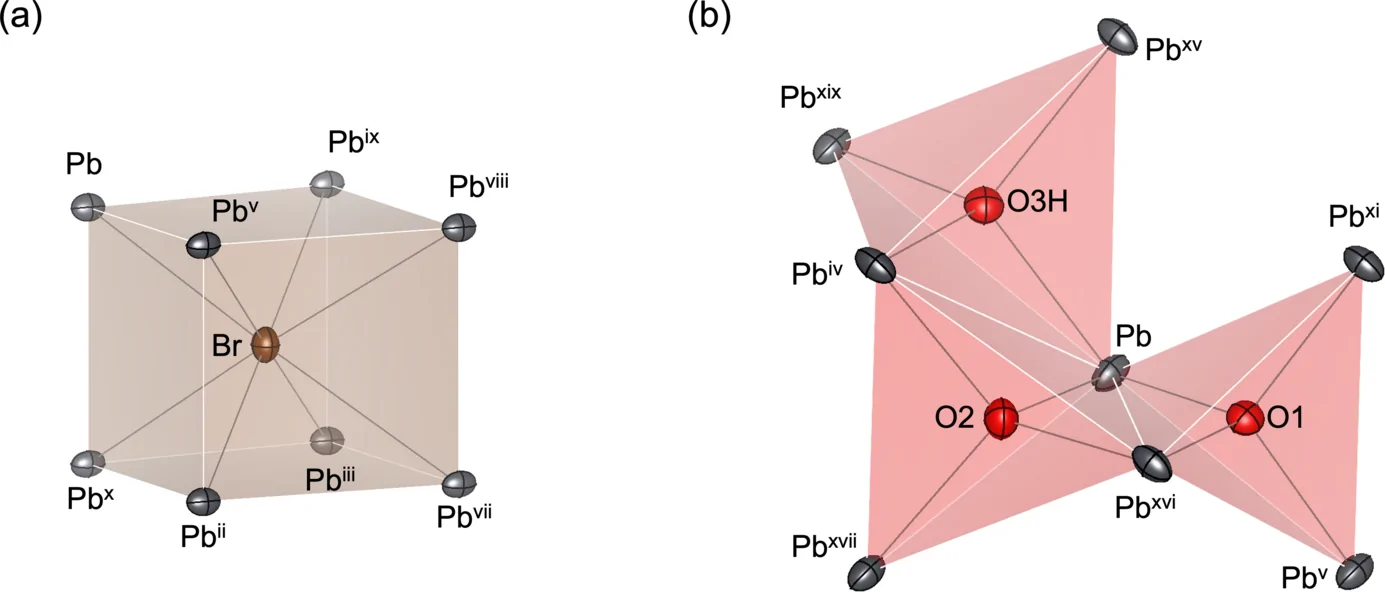
Coordination environments of the (*a*) Br and (*b*) O atoms. Ellipsoids are drawn at the 50% probability level. [Symmetry codes: (ii) −*x* + 1, −*y* + 1, −*z*; (iii) −*x*, −*y* + 1, −*z*; (iv) *y*, −*x*, −*z* + 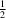; (v) −*x* + 1, −*y* + 1, *z*; (vi) *y*, −*x* + 1, *z* − 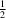; (vii) *x*, *y* + 1, −*z*; (viii) *x*, *y* + 1, *z*; (ix) −*x*, −*y* + 1, *z*; (*x*) *x*, *y*, −*z*; (xi) *y*, −*x* + 1, −*z* + 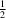; (xv) −*y*, *x*, −*z* + 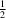; (xvi) −*y* + 1, *x*, −*z* + 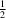; (xvii) −*x* + 1, −*y*, *z*; (xix) −*x*, −*y*, *z*.]

The title compound crystallizes in the tetra­gonal space group *P*4_2_/*mcm*. We note that the crystal structure of Pb_2_O(OH)Br is different from the ones of Pb_2_O(OH)Cl (space group *Pnma*) and Pb_2_O(OH)I (space group *C*2/*m*). The asymmetric unit contains one Pb site, three O sites (O1, O2 and O3), and one Br site. The divalent Pb cation is coordinated in a square-anti­prismatic mode by four O and four Br atoms (Fig. 1 ▸ and Table 1 ▸). The O_4_ and Br_4_ squares are parallel, rotated by 45° relative to each other, with the Br_4_ square having an edge length approximately √2 times that of the O_4_ square. The face-sharing square anti­prisms form square layers in the *ab* plane (Fig. 2 ▸). These layers with opposite orientations are alternately stacked along the *c* axis. Consequently, a Br square layer and an O square layer are formed alternately along the *c* axis with 45° relative rotation. The Br atom is surrounded by eight Pb cations in a distorted cubic arrangement, while each of the O atoms is surrounded by four Pb cations in a distorted tetra­hedral mode (Fig. 3 ▸).

Charge neutrality requires the presence of one H atom per formula unit, although the H atom could not be located from difference Fourier maps owing to the presence of the heavy Pb atom. Bond-valence-sum (BVS) calculations (Brown, 2002 ▸; Brese & O’Keeffe, 1991 ▸) were therefore carried out to identify the hydroxide site (Table 2 ▸). Consistent with the longest Pb—O3 bond length, O3 exhibits the lowest BVS value, supporting its assignment as the hydroxide site. We note that the O2 site cannot be assigned as the hydroxide site because its multiplicity is twice that of the O1 and O3 sites. The off-centered coordination around the Pb^2+^ cation reflects the stereochemical activity of its 6*s*^2^ electron lone pair.

## Synthesis and crystallization

Single crystals of Pb_2_O(OH)Br were obtained serendipitously during attempts to synthesize the Br analogues of diaboleite, Pb_2_CuCl_2_(OH)_4_, and cumengeite, Pb_21_Cu_20_Cl_42_(OH)_40_·6H_2_O, (Alun Humphreys *et al.*, 1980 ▸) under hydro­thermal conditions. Pb wire (0.6 g, Nilaco), CuBr_2_ (0.2 g, FUJIFILM Wako), and 5 wt% aqueous ammonia (8 ml) were placed in a polymer-lined stainless-steel autoclave and heated at 533 K for 24 h. Yellow, plate-like crystals grew on the surface of the Pb wire, and a suitable crystal was selected for the single-crystal X-ray diffraction measurements.

## Refinement

Details of data collection and structure refinement are given in Table 3 ▸. The largest residual electron-density peaks are located within 0.9 Å of the Pb atom and are attributed to Fourier termination errors associated with the heavy Pb atom.

## Supplementary Material

Crystal structure: contains datablock(s) I. DOI: 10.1107/S241431462600790X/wm4254sup1.cif

Structure factors: contains datablock(s) I. DOI: 10.1107/S241431462600790X/wm4254Isup2.hkl

CCDC reference: 2578647

Additional supporting information:  crystallographic information; 3D view; checkCIF report

## Figures and Tables

**Table 1:** Selected bond lengths (Å)

Pb—Br^i^	3.3741 (15)	Pb—O1	2.3101 (6)
Pb—Br	3.3741 (15)	Pb—O2^iv^	2.4291 (3)
Pb—Br^ii^	3.6154 (15)	Pb—O2	2.4291 (3)
Pb—Br^iii^	3.6154 (15)	Pb—O3*H*	2.5426 (6)

Symmetry codes: (i) 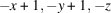; (ii) 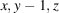; (iii) 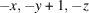; (iv) 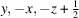.

**Table 2:** Atomic sites (multiplicity and Wyckoff letter), site symmetries, and bond-valence sums (in valence units)

Pb (8 *o*)	*m*	2.16
Br (4 *j*)	*mm*2	0.84
O1 (2 *d*)	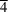 2*m*	2.34
O2 (4 *e*)	222	1.70
O3*H* (2 *b*)	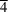 2*m*	1.25

**Table 3:** Experimental details

Crystal data	
Chemical formula	Pb_2_O(OH)Br
*M*_r_	526.29
Crystal system, space group	Tetragonal, *P*4_2_/*mcm*
Temperature (K)	296
*a*, *c* (Å)	5.8161 (3), 12.8870 (13)
*V* (Å^3^)	435.93 (6)
*Z*	4
Radiation type	Mo *K*α
μ (mm^−1^)	86.13
Crystal size (mm)	0.10 × 0.08 × 0.01

Data collection	
Diffractometer	XtaLAB AFC11 (RCD3): quarter-chi single
Absorption correction	Multi-scan (*CrysAlis PRO*; Rigaku OD, 2023 ▸)
*T*_min_, *T*_max_	0.542, 1.000
No. of measured, independent and observed [*I* > 2σ(*I*)] reflections	9139, 705, 460
*R*_int_	0.062
(sin θ/λ)_max_ (Å^−1^)	0.882

Refinement	
*R*[*F*^2^ > 2σ(*F*^2^)], *wR*(*F*^2^), *S*	0.044, 0.131, 1.12
No. of reflections	705
No. of parameters	18
H-atom treatment	H-atom parameters not defined
Δρ_max_, Δρ_min_ (e Å^−3^)	6.55, −4.22

Computer programs: *CrysAlis PRO* (Rigaku OD, 2023 ▸), *SHELXT* (Sheldrick, 2015*a* ▸), *SHELXL* (Sheldrick, 2015*b* ▸), *VESTA* (Momma & Izumi, 2011 ▸), *OLEX2* (Dolomanov *et al.*, 2009 ▸) and *publCIF* (Westrip, 2010 ▸).

## full crystallographic data

### Dilead(II) oxyhydroxide bromide Crystal data

**Table:**

Pb_2_O(OH)Br	*D*_x_ = 8.019 Mg m^−^^3^
*M**_r_* = 526.29	Mo *K*α radiation, λ = 0.71073 Å
Tetragonal, *P*4_2_/*m**c**m*	Cell parameters from 2463 reflections
*a* = 5.8161 (3) Å	θ = 3.1–34.3°
*c* = 12.8870 (13) Å	µ = 86.13 mm^−^^1^
*V* = 435.93 (6) Å^3^	*T* = 296 K
*Z* = 4	Plate, yellow
*F*(000) = 860	0.10 × 0.08 × 0.01 mm

### Dilead(II) oxyhydroxide bromide Data collection

**Table:**

XtaLAB AFC11 (RCD3): quarter-chi single diffractometer	705 independent reflections
Radiation source: Rotating-anode X-ray tube, Rigaku (Mo) X-ray Source	460 reflections with *I* > 2σ(*I*)
Mirror monochromator	*R*_int_ = 0.062
Detector resolution: 10.0000 pixels mm^-1^	θ_max_ = 38.8°, θ_min_ = 3.2°
ω scans	*h* = −8→10
Absorption correction: multi-scan (CrysAlisPro; Rigaku OD, 2023)	*k* = −10→10
*T*_min_ = 0.542, *T*_max_ = 1.000	*l* = −22→18
9139 measured reflections	

### Dilead(II) oxyhydroxide bromide Refinement

**Table:**

Refinement on *F*^2^	0 restraints
Least-squares matrix: full	Primary atom site location: dual
*R*[*F*^2^ > 2σ(*F*^2^)] = 0.044	H-atom parameters not defined
*wR*(*F*^2^) = 0.131	*w* = 1/[σ^2^(*F*_o_^2^) + (0.0369*P*)^2^ + 26.4764*P*] where *P* = (*F*_o_^2^ + 2*F*_c_^2^)/3
*S* = 1.12	(Δ/σ)_max_ < 0.001
705 reflections	Δρ_max_ = 6.55 e Å^−^^3^
18 parameters	Δρ_min_ = −4.22 e Å^−^^3^

### Dilead(II) oxyhydroxide bromide Special details

**Table:**

Geometry. All esds (except the esd in the dihedral angle between two l.s. planes) are estimated using the full covariance matrix. The cell esds are taken into account individually in the estimation of esds in distances, angles and torsion angles; correlations between esds in cell parameters are only used when they are defined by crystal symmetry. An approximate (isotropic) treatment of cell esds is used for estimating esds involving l.s. planes.

### Dilead(II) oxyhydroxide bromide Fractional atomic coordinates and isotropic or equivalent isotropic displacement parameters (Å^2^)

**Table:**

	*x*	*y*	*z*	*U*_iso_*/*U*_eq_	
Pb	0.26668 (8)	0.26668 (8)	0.15022 (4)	0.01991 (19)	
Br	0.2582 (3)	0.7418 (3)	0.000000	0.0233 (4)	
O1	0.500000	0.500000	0.250000	0.018 (7)	
O2	0.000000	0.500000	0.250000	0.023 (8)	
O3H	0.000000	0.000000	0.250000	0.020 (7)	

### Dilead(II) oxyhydroxide bromide Atomic displacement parameters (Å^2^)

**Table:**

	*U*^11^	*U*^22^	*U*^33^	*U*^12^	*U*^13^	*U*^23^
Pb	0.0215 (2)	0.0215 (2)	0.0168 (3)	−0.0095 (3)	−0.00071 (12)	−0.00071 (12)
Br	0.0204 (6)	0.0204 (6)	0.0290 (11)	0.0022 (15)	0.000	0.000
O1	0.020 (10)	0.020 (10)	0.015 (16)	0.000	0.000	0.000
O2	0.016 (10)	0.024 (12)	0.03 (2)	0.000	0.000	0.000
O3H	0.022 (11)	0.022 (11)	0.017 (17)	0.000	0.000	0.000

### Dilead(II) oxyhydroxide bromide Geometric parameters (Å, º)

**Table:**

Pb—Br^i^	3.3741 (15)	Pb—O1	2.3101 (6)
Pb—Br	3.3741 (15)	Pb—O2^iv^	2.4291 (3)
Pb—Br^ii^	3.6154 (15)	Pb—O2	2.4291 (3)
Pb—Br^iii^	3.6154 (15)	Pb—O3H	2.5426 (6)
			
Br—Pb—Br^i^	72.22 (6)	Pb—O1—Pb^ix^	58.337 (6)
Br^iii^—Pb—Br^ii^	71.96 (6)	Pb^xiv^—O1—Pb^xvii^	94.056 (3)
Br—Pb—Br^iii^	72.039 (8)	Pb^xv^—O1—Pb^viii^	94.056 (4)
Br^i^—Pb—Br^ii^	72.039 (8)	Pb—O2—Pb^xi^	100.64 (3)
Br—Pb—Br^ii^	112.590 (16)	Pb^xviii^—O2—Pb^xix^	51.065 (2)
Br^i^—Pb—Br^iii^	112.590 (16)	Pb^xviii^—O2—Pb^xx^	92.740 (7)
O1—Pb—Br	81.191 (16)	Pb^viii^—O2—Pb^iv^	143.039 (7)
O1—Pb—Br^i^	81.191 (16)	Pb^xvi^—O2—Pb^viii^	92.740 (7)
O1—Pb—Br^ii^	143.33 (3)	Pb^ix^—O2—Pb^xx^	55.085 (16)
O1—Pb—Br^iii^	143.33 (3)	Pb^xv^—O2—Pb^iv^	63.525 (18)
O1—Pb—O2	75.655 (14)	Pb—O2—Pb^xv^	112.08 (3)
O1—Pb—O2^iv^	75.655 (14)	Pb^xi^—O2—Pb^viii^	55.085 (16)
O1—Pb—O3H	115.80 (2)	Pb^xviii^—O2—Pb^viii^	118.901 (6)
O2—Pb—Br^iii^	75.63 (3)	Pb—O2—Pb^xx^	148.277 (12)
O2^iv^—Pb—Br^ii^	75.63 (3)	Pb^xi^—O2—Pb^xv^	116.07 (2)
O2^iv^—Pb—Br	146.59 (3)	Pb^xix^—O2—Pb^xx^	143.039 (7)
O2—Pb—Br^i^	146.59 (3)	Pb^xi^—O2—Pb^iv^	99.647 (17)
O2—Pb—Br^ii^	138.29 (3)	Pb^v^—O2—Pb^iv^	92.740 (7)
O2^iv^—Pb—Br^iii^	138.29 (3)	Pb—O2—Pb^ix^	116.07 (2)
O2—Pb—Br	80.62 (3)	Pb^xxi^—O2—Pb^xix^	92.740 (7)
O2^iv^—Pb—Br^i^	80.62 (3)	Pb—O2—Pb^viii^	99.647 (17)
O2^iv^—Pb—O2	115.67 (2)	Pb^ix^—O2—Pb^viii^	63.525 (19)
O2—Pb—O3H	71.552 (14)	Pb^xi^—O2—Pb^ix^	112.08 (3)
O2^iv^—Pb—O3H	71.552 (14)	Pb^xxi^—O2—Pb^viii^	95.585 (7)
O3H—Pb—Br^ii^	75.368 (15)	Pb^xix^—O2—Pb^viii^	149.527 (14)
O3H—Pb—Br^iii^	75.368 (15)	Pb^xv^—O2—Pb^xx^	99.647 (17)
O3H—Pb—Br	141.34 (3)	Pb^xv^—O2—Pb^ix^	100.64 (3)
O3H—Pb—Br^i^	141.34 (3)	Pb^v^—O2—Pb^xx^	95.585 (8)
Pb^v^—Br—Pb^vi^	124.37 (4)	Pb^xxi^—O2—Pb^xx^	51.065 (2)
Pb^i^—Br—Pb^vii^	72.180 (8)	Pb—O2—Pb^iv^	55.085 (16)
Pb^viii^—Br—Pb^ix^	74.70 (4)	Pb—O2—Pb^xviii^	106.011 (17)
Pb—Br—Pb^vi^	124.37 (4)	Pb^xviii^—O2—Pb^iv^	95.585 (7)
Pb^x^—Br—Pb^ix^	107.961 (8)	Pb^xvi^—O2—Pb^iv^	51.065 (2)
Pb^iii^—Br—Pb^vi^	58.241 (14)	Pb^xix^—O2—Pb^iv^	48.630 (9)
Pb^x^—Br—Pb^vii^	112.591 (17)	Pb^xi^—O2—Pb^xviii^	153.350 (11)
Pb^ix^—Br—Pb^vi^	122.98 (3)	Pb^xi^—O2—Pb^v^	53.030 (17)
Pb—Br—Pb^viii^	112.590 (16)	Pb^v^—O2—Pb^xix^	118.901 (6)
Pb^v^—Br—Pb^xi^	54.357 (14)	Pb^xv^—O2—Pb^v^	153.350 (11)
Pb^i^—Br—Pb^ix^	176.910 (14)	Pb^xv^—O2—Pb^xviii^	53.030 (17)
Pb—Br—Pb^xi^	54.357 (14)	Pb^ix^—O2—Pb^v^	106.011 (17)
Pb^viii^—Br—Pb^iii^	108.04 (6)	Pb^xv^—O2—Pb^viii^	148.277 (12)
Pb^x^—Br—Pb^viii^	176.910 (15)	Pb^xviii^—O2—Pb^v^	147.940 (14)
Pb—Br—Pb^vii^	176.910 (15)	Pb^ix^—O2—Pb^xviii^	55.377 (19)
Pb^v^—Br—Pb^viii^	72.179 (8)	Pb^xvi^—O2—Pb^v^	47.339 (10)
Pb^i^—Br—Pb^viii^	107.961 (8)	Pb^v^—O2—Pb^viii^	51.065 (2)
Pb^iii^—Br—Pb^vii^	74.70 (4)	Pb—O2—Pb^xxi^	153.350 (11)
Pb^ix^—Br—Pb^xi^	58.241 (14)	Pb—O2—Pb^xvi^	53.029 (17)
Pb^x^—Br—Pb^xi^	124.37 (4)	Pb^xi^—O2—Pb^xxi^	106.011 (17)
Pb^vii^—Br—Pb^ix^	108.04 (6)	Pb^xi^—O2—Pb^xx^	63.525 (19)
Pb^v^—Br—Pb^iii^	176.910 (15)	Pb^xv^—O2—Pb^xxi^	55.377 (19)
Pb^x^—Br—Pb^vi^	54.357 (14)	Pb^xi^—O2—Pb^xvi^	55.377 (19)
Pb^i^—Br—Pb^xi^	124.37 (4)	Pb^ix^—O2—Pb^xxi^	53.030 (17)
Pb^i^—Br—Pb^vi^	54.357 (14)	Pb^xvi^—O2—Pb^xx^	118.901 (6)
Pb—Br—Pb^iii^	107.961 (8)	Pb^xviii^—O2—Pb^xxi^	47.339 (9)
Pb^viii^—Br—Pb^vi^	122.98 (3)	Pb^xv^—O2—Pb^xvi^	106.011 (17)
Pb^iii^—Br—Pb^xi^	122.98 (3)	Pb^xvi^—O2—Pb^xxi^	147.940 (14)
Pb^x^—Br—Pb^v^	107.78 (6)	Pb^viii^—O2—Pb^xx^	48.630 (8)
Pb^v^—Br—Pb^ix^	112.591 (17)	Pb^v^—O2—Pb^xxi^	146.116 (7)
Pb^x^—Br—Pb^i^	69.33 (4)	Pb^ix^—O2—Pb^xvi^	153.350 (11)
Pb^v^—Br—Pb^vii^	107.961 (8)	Pb—O2—Pb^xix^	63.525 (19)
Pb^v^—Br—Pb^i^	70.02 (4)	Pb^ix^—O2—Pb^iv^	148.277 (12)
Pb^i^—Br—Pb^iii^	112.591 (17)	Pb^xi^—O2—Pb^xix^	148.277 (12)
Pb^x^—Br—Pb	70.02 (4)	Pb^xviii^—O2—Pb^xvi^	146.116 (7)
Pb—Br—Pb^ix^	72.179 (8)	Pb^xv^—O2—Pb^xix^	55.085 (16)
Pb^v^—Br—Pb	69.33 (4)	Pb^xxi^—O2—Pb^iv^	118.901 (6)
Pb^x^—Br—Pb^iii^	72.180 (8)	Pb^ix^—O2—Pb^xix^	99.647 (17)
Pb^i^—Br—Pb	107.78 (6)	Pb—O2—Pb^v^	55.377 (19)
Pb^viii^—Br—Pb^vii^	64.75 (3)	Pb^xvi^—O2—Pb^xix^	95.585 (7)
Pb^vi^—Br—Pb^xi^	178.24 (6)	Pb^xx^—O2—Pb^iv^	149.527 (14)
Pb^iii^—Br—Pb^ix^	64.75 (3)	Pb—O3H—Pb^xix^	119.24 (3)
Pb^viii^—Br—Pb^xi^	58.241 (14)	Pb^ix^—O3H—Pb^xvii^	121.353 (13)
Pb^vii^—Br—Pb^vi^	58.241 (14)	Pb^xvi^—O3H—Pb^ix^	94.262 (4)
Pb^vii^—Br—Pb^xi^	122.98 (3)	Pb^xviii^—O3H—Pb^xvii^	148.360 (14)
Pb—O1—Pb^v^	112.35 (3)	Pb^xi^—O3H—Pb^xviii^	94.262 (4)
Pb^iv^—O1—Pb^viii^	147.401 (13)	Pb^iv^—O3H—Pb^ix^	152.875 (9)
Pb^xii^—O1—Pb^xiii^	94.056 (4)	Pb^xv^—O3H—Pb^xvii^	152.875 (9)
Pb^xiii^—O1—Pb^viii^	116.547 (13)	Pb—O3H—Pb^xv^	104.820 (12)
Pb^xiv^—O1—Pb^xv^	149.152 (13)	Pb^xix^—O3H—Pb^xviii^	60.734 (7)
Pb^xi^—O1—Pb^xiii^	148.721 (10)	Pb^xvi^—O3H—Pb^xviii^	141.606 (14)
Pb^xvi^—O1—Pb^viii^	148.721 (10)	Pb—O3H—Pb^ix^	60.734 (7)
Pb—O1—Pb^xvi^	108.051 (15)	Pb^xix^—O3H—Pb^xv^	104.820 (12)
Pb^v^—O1—Pb^xv^	148.721 (10)	Pb^ii^—O3H—Pb^ix^	148.360 (14)
Pb^xii^—O1—Pb^xv^	116.547 (13)	Pb^xix^—O3H—Pb^xvii^	102.305 (10)
Pb—O1—Pb^xiii^	103.228 (10)	Pb^xxii^—O3H—Pb^xvii^	94.262 (4)
Pb^v^—O1—Pb^xvi^	108.051 (15)	Pb—O3H—Pb^iv^	104.820 (12)
Pb^xvii^—O1—Pb^xiii^	53.957 (13)	Pb^xxiii^—O3H—Pb^ii^	94.262 (4)
Pb^v^—O1—Pb^viii^	58.337 (6)	Pb—O3H—Pb^xviii^	102.305 (10)
Pb^ix^—O1—Pb^viii^	53.957 (13)	Pb^iv^—O3H—Pb^xviii^	152.875 (9)
Pb—O1—Pb^xi^	108.051 (15)	Pb^xix^—O3H—Pb^iv^	104.820 (12)
Pb^iv^—O1—Pb^xvii^	45.493 (14)	Pb^xxiii^—O3H—Pb^xviii^	50.571 (15)
Pb—O1—Pb^xv^	56.043 (19)	Pb^ii^—O3H—Pb^xviii^	121.353 (13)
Pb^xi^—O1—Pb^xv^	58.337 (6)	Pb^xv^—O3H—Pb^ix^	52.101 (18)
Pb^v^—O1—Pb^xi^	108.051 (15)	Pb^xv^—O3H—Pb^iv^	119.24 (3)
Pb^iv^—O1—Pb^xv^	53.957 (13)	Pb^xxii^—O3H—Pb^ix^	141.606 (14)
Pb^xvii^—O1—Pb^xv^	94.056 (4)	Pb^xxiii^—O3H—Pb^ix^	94.262 (4)
Pb^xvi^—O1—Pb^xiii^	56.044 (19)	Pb—O3H—Pb^xvii^	60.734 (7)
Pb^xvi^—O1—Pb^xi^	112.35 (3)	Pb—O3H—Pb^xvi^	52.101 (18)
Pb^ix^—O1—Pb^xiii^	149.152 (13)	Pb^xvi^—O3H—Pb^xvii^	50.571 (15)
Pb^iv^—O1—Pb^xiii^	94.056 (4)	Pb^xi^—O3H—Pb^xvii^	94.262 (4)
Pb—O1—Pb^viii^	103.228 (10)	Pb^ii^—O3H—Pb^xvii^	48.014 (14)
Pb—O1—Pb^xii^	148.721 (10)	Pb^xix^—O3H—Pb^xvi^	152.875 (9)
Pb^xii^—O1—Pb^viii^	45.493 (14)	Pb^xix^—O3H—Pb^xxii^	52.101 (18)
Pb^xiv^—O1—Pb^viii^	94.056 (4)	Pb^xxii^—O3H—Pb^ii^	50.571 (15)
Pb^xi^—O1—Pb^xvii^	148.721 (10)	Pb^xv^—O3H—Pb^xxii^	102.305 (10)
Pb^v^—O1—Pb^xii^	56.044 (19)	Pb^xv^—O3H—Pb^xvi^	102.305 (10)
Pb^v^—O1—Pb^ix^	103.228 (10)	Pb^iv^—O3H—Pb^xxii^	60.734 (7)
Pb^ix^—O1—Pb^xvii^	116.547 (13)	Pb^xv^—O3H—Pb^xviii^	52.101 (18)
Pb^xvi^—O1—Pb^ix^	148.721 (10)	Pb^xvi^—O3H—Pb^xxii^	121.353 (13)
Pb^xvi^—O1—Pb^xii^	103.228 (10)	Pb^iv^—O3H—Pb^xvi^	60.734 (7)
Pb^xi^—O1—Pb^ix^	56.044 (19)	Pb^xi^—O3H—Pb^xxii^	148.360 (14)
Pb^xvi^—O1—Pb^xv^	103.228 (10)	Pb^xxii^—O3H—Pb^xviii^	94.262 (4)
Pb^xii^—O1—Pb^ix^	94.056 (3)	Pb—O3H—Pb^xxiii^	152.876 (9)
Pb^xi^—O1—Pb^xii^	58.337 (6)	Pb—O3H—Pb^xi^	52.101 (18)
Pb^xiv^—O1—Pb^ix^	147.401 (13)	Pb^xix^—O3H—Pb^xxiii^	52.101 (18)
Pb^ix^—O1—Pb^xv^	45.493 (14)	Pb^xix^—O3H—Pb^ix^	102.305 (10)
Pb—O1—Pb^iv^	56.043 (19)	Pb^xv^—O3H—Pb^xxiii^	60.734 (7)
Pb—O1—Pb^xiv^	148.721 (10)	Pb^xix^—O3H—Pb^xi^	152.875 (9)
Pb^v^—O1—Pb^iv^	148.721 (10)	Pb^iv^—O3H—Pb^xxiii^	102.305 (10)
Pb^v^—O1—Pb^xiii^	58.337 (6)	Pb^xi^—O3H—Pb^ix^	50.571 (15)
Pb^xvi^—O1—Pb^iv^	58.337 (6)	Pb^xvi^—O3H—Pb^xxiii^	148.360 (14)
Pb^v^—O1—Pb^xiv^	56.044 (19)	Pb^xv^—O3H—Pb^xi^	60.734 (7)
Pb^xi^—O1—Pb^iv^	103.228 (10)	Pb^xi^—O3H—Pb^xxiii^	121.353 (13)
Pb^xiv^—O1—Pb^xiii^	45.493 (14)	Pb^xviii^—O3H—Pb^ix^	48.014 (14)
Pb^xii^—O1—Pb^iv^	149.152 (13)	Pb^xxii^—O3H—Pb^xxiii^	48.014 (14)
Pb^xvi^—O1—Pb^xiv^	58.337 (6)	Pb^iv^—O3H—Pb^xi^	102.305 (10)
Pb^xiv^—O1—Pb^iv^	116.547 (13)	Pb—O3H—Pb^ii^	102.305 (10)
Pb^xv^—O1—Pb^xiii^	147.401 (13)	Pb^iv^—O3H—Pb^xvii^	52.101 (18)
Pb^ix^—O1—Pb^iv^	94.056 (4)	Pb^xix^—O3H—Pb^ii^	60.734 (7)
Pb^xi^—O1—Pb^xiv^	103.228 (10)	Pb^xvi^—O3H—Pb^xi^	48.014 (14)
Pb—O1—Pb^xvii^	58.337 (6)	Pb^xv^—O3H—Pb^ii^	152.875 (9)
Pb^xi^—O1—Pb^viii^	56.044 (19)	Pb^xxiii^—O3H—Pb^xvii^	141.606 (14)
Pb^v^—O1—Pb^xvii^	103.228 (10)	Pb^iv^—O3H—Pb^ii^	52.101 (18)
Pb^xii^—O1—Pb^xiv^	53.957 (13)	Pb—O3H—Pb^xxii^	152.876 (9)
Pb^xvi^—O1—Pb^xvii^	56.044 (19)	Pb^xvi^—O3H—Pb^ii^	94.262 (4)
Pb^xvii^—O1—Pb^viii^	149.152 (13)	Pb^xi^—O3H—Pb^ii^	141.606 (14)
Pb^xii^—O1—Pb^xvii^	147.401 (13)		

Symmetry codes: (i) −*x*+1, −*y*+1, −*z*; (ii) *x*, *y*−1, *z*; (iii) −*x*, −*y*+1, −*z*; (iv) *y*, −*x*, −*z*+1/2; (v) −*x*+1, −*y*+1, *z*; (vi) *y*, −*x*+1, *z*−1/2; (vii) *x*, *y*+1, −*z*; (viii) *x*, *y*+1, *z*; (ix) −*x*, −*y*+1, *z*; (x) *x*, *y*, −*z*; (xi) *y*, −*x*+1, −*z*+1/2; (xii) −*y*+1, *x*+1, −*z*+1/2; (xiii) *x*+1, *y*, *z*; (xiv) *y*+1, −*x*+1, −*z*+1/2; (xv) −*y*, *x*, −*z*+1/2; (xvi) −*y*+1, *x*, −*z*+1/2; (xvii) −*x*+1, −*y*, *z*; (xviii) *x*−1, *y*, *z*; (xix) −*x*, −*y*, *z*; (xx) −*y*, *x*+1, −*z*+1/2; (xxi) *y*−1, −*x*+1, −*z*+1/2; (xxii) −*y*, *x*−1, −*z*+1/2; (xxiii) *y*−1, −*x*, −*z*+1/2.

## References

[bb1] Alun Humphreys, D., Thomas, J. H., Williams, P. A. & Symes, R. F. (1980). *Miner. Mag.* **43**, 901–904.

[bb2] Brese, N. E. & O’Keeffe, M. (1991). *Acta Cryst.* B**47**, 192–197.

[bb3] Brown, I. D. (2002). *The Chemical Bond in Inorganic Chemistry: The Bond Valence Model*. Oxford University Press.

[bb4] Dolomanov, O. V., Bourhis, L. J., Gildea, R. J., Howard, J. A. K. & Puschmann, H. (2009). *J. Appl. Cryst.* **42**, 339–341.

[bb5] Krivovichev, S. V. & Burns, P. C. (2001). *Solid State Sci.* **3**, 455–459.

[bb6] Momma, K. & Izumi, F. (2011). *J. Appl. Cryst.* **44**, 1272–1276.

[bb7] Rigaku OD (2023). *CrysAlis PRO*. Rigaku Oxford Diffraction, Yarnton, England.

[bb8] Sheldrick, G. M. (2015*a*). *Acta Cryst.* A**71**, 3–8.

[bb9] Sheldrick, G. M. (2015*b*). *Acta Cryst.* C**71**, 3–8.

[bb10] Siidra, O. I., Zenko, D. Y., Suknotova, A. N. & Krivovichev, S. V. (2013). *Miner. Mag.* **77**, 3239–3248.

[bb11] Turner, R. W., Siidra, O. I., Rumsey, M. S., Polekhovsky, Y. S., Kretser, Y. L., Krivovichev, S. V., Spratt, J. & Stanley, C. J. (2015). *Miner. Mag.* **79**, 1203–1211.

[bb12] Westrip, S. P. (2010). *J. Appl. Cryst.* **43**, 920–925.

